# Author Correction: Adaptive gene loss in the common bean pan-genome during range expansion and domestication

**DOI:** 10.1038/s41467-024-52864-8

**Published:** 2024-10-08

**Authors:** Gaia Cortinovis, Leonardo Vincenzi, Robyn Anderson, Giovanni Marturano, Jacob Ian Marsh, Philipp Emanuel Bayer, Lorenzo Rocchetti, Giulia Frascarelli, Giovanna Lanzavecchia, Alice Pieri, Andrea Benazzo, Elisa Bellucci, Valerio Di Vittori, Laura Nanni, Juan José Ferreira Fernández, Marzia Rossato, Orlando Mario Aguilar, Peter Laurent Morrell, Monica Rodriguez, Tania Gioia, Kerstin Neumann, Juan Camilo Alvarez Diaz, Ariane Gratias, Christophe Klopp, Elena Bitocchi, Valérie Geffroy, Massimo Delledonne, David Edwards, Roberto Papa

**Affiliations:** 1 Department of Agricultural, Food and Environmental Sciences, Marche Polytechnic University, 60131 Ancona, Italy; 2 Department of Biotechnology, University of Verona, 37134 Verona, Italy; 3 Centre for Applied Bioinformatics and School of Biological Sciences, University of Western Australia, Perth, WA 6009 Australia; 4 Department of Life Sciences and Biotechnology, University of Ferrara, 44100 Ferrara, Italy; 5 Regional Service for Agrofood Research and Development (SERIDA), Ctra AS-267 PK 19, 33300 Asturias, Spain; 6 Genartis s.r.l, 37126 Verona, Italy; 7 Institute of Biotechnology and Molecular Biology, UNLP-CONICET, CCT La Plata, La Plata, Argentina; 8 Department of Agronomy and Plant Genetics, University of Minnesota, St. Paul, MN 55108-6026 USA; 9 Department of Agriculture, University of Sassari, 07100 Sassari, Italy; 10 CBV—Centro per la Conservazione e Valorizzazione della Biodiversità Vegetale, University of Sassari, 07041 Alghero, Italy; 11 School of Agricultural, Forestry, Food and Environmental Sciences, University of Basilicata, 85100 Potenza, Italy; 12 Leibniz Institute of Plant Genetics and Crop Plant Research (IPK), 06466 Seeland, Germany; 13 CNRS, INRAE, Institute of Plant Sciences Paris-Saclay (IPS2), University of Evry, University Paris-Saclay, 91405 Orsay, France; 14 INRAE, Genotoul Bioinformatics Platform, Applied Mathematics and Informatics of Toulouse, Sigenae, MIAT, UR875 Castanet Tolosan, France

**Keywords:** Plant evolution, Agricultural genetics, Plant domestication, Structural variation

Correction to: *Nature Communications* 10.1038/s41467-024-51032-2, published online 07 August 2024

Both HTML and PDF versions of this Article previously published did not acknowledge Valérie Geffroy (valerie.geffroy@universite-paris-saclay.fr), Massimo Delledonne (massimo.delledonne@univr.it), and David Edwards (Dave.Edwards@uwa.edu.au) as corresponding authors. This has been corrected in both the PDF and HTML versions of the Article.

