# Supplementary material for: Adaptive gene loss in the common bean pan-genome during range expansion and domestication
Source: Nat Commun. 2024 Aug 7;15:6698. doi: 10.1038/s41467-024-51032-2 (PMC11303546; doi:10.1038/s41467-024-51032-2)
Supplement: Supplementary file 1 — Supplementary Information [file 41467_2024_51032_MOESM1_ESM.pdf]

Adaptive gene loss in the common bean pan-genome during range expansion and domestication

Cortinovis *et al.*

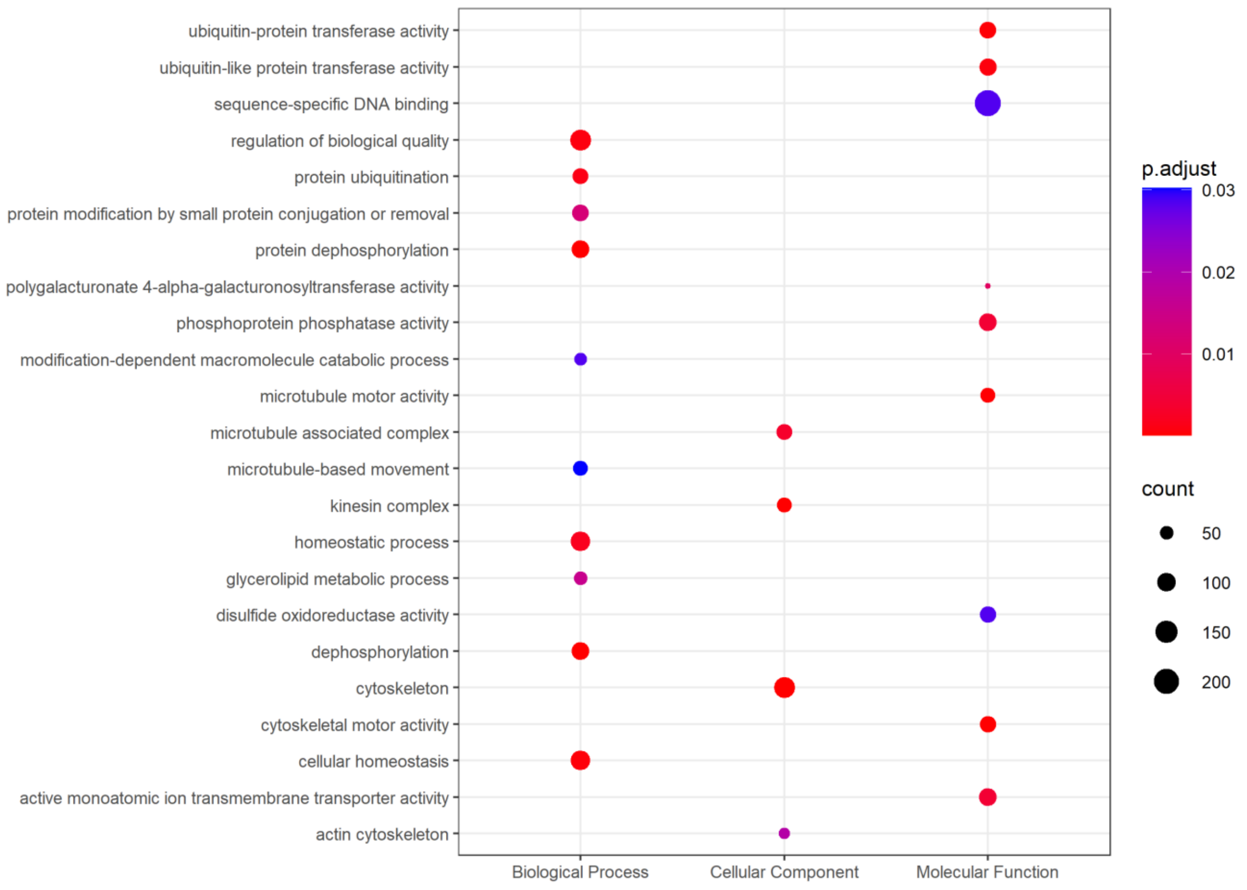

Supplementary Fig. 1. Characterization of the common bean pan-genome. Dot plot showing the GO enrichment analysis of core genes. The GO enrichment analysis was performed using the enricher function from the cluster Profiler R package, which employs a hypergeometric test to identify over-represented GO terms in the core genes. To control for multiple comparisons, the *p*-values were adjusted using the Benjamini-Hochberg (BH) method.

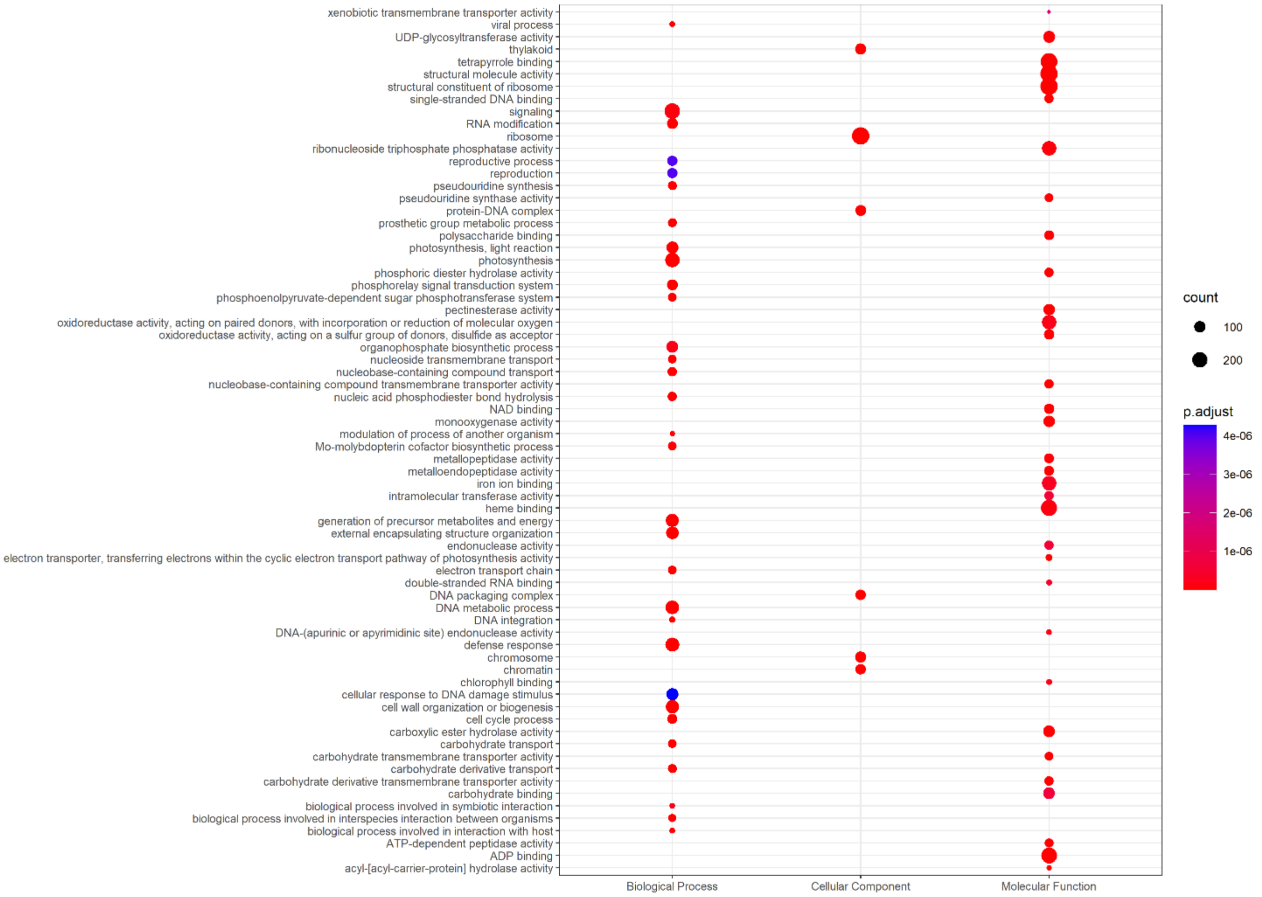

Supplementary Fig. 2. Characterization of the common bean pan-genome. Dot plot showing the GO enrichment analysis of PAVs (presence/absence variations). Only the top 70 enriched GO categories (based on *p*-values) are shown. The GO enrichment analysis was performed using the enricher function from the clusterProfiler R package, which employs a hypergeometric test to identify over-represented GO terms in the PAV genes. To control for multiple comparisons, the *p*-values were adjusted using the Benjamini-Hochberg (BH) method.

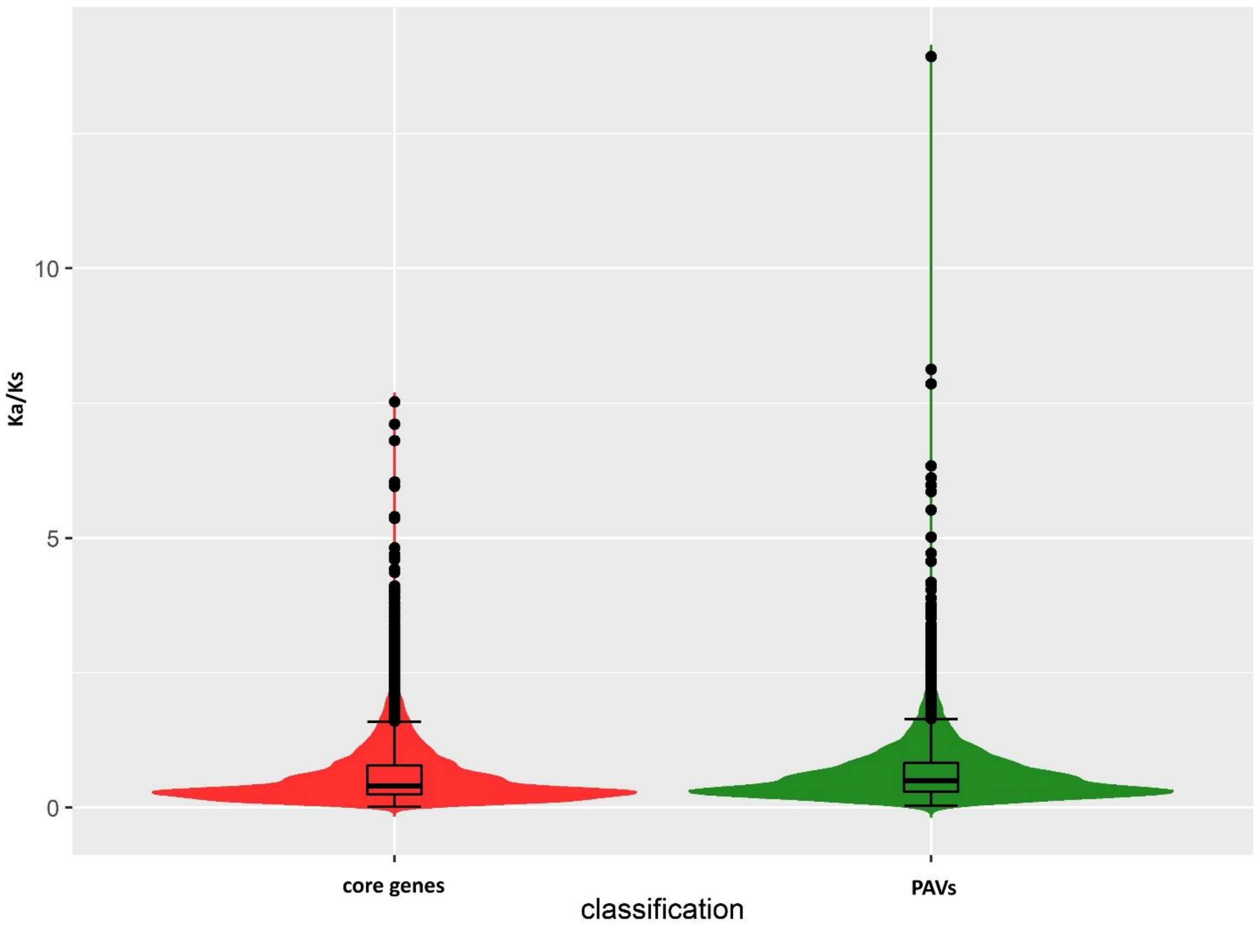

Supplementary Fig. 3. Characterization of the common bean pan-genome. Violin plots showing the analysis of variance (ANOVA) related to the ratio of non-synonymous to synonymous mutations (Ka/Ks) in core genes and PAVs. Sample sizes (n) for each category are as follows: core n=16,264, PAVs n=4,968. Statistical significance was determined by applying a Kruskal-Wallis chi-squared test ($p < 2.2 \times 10^{-16}$). Source data are provided as a Source Data file.

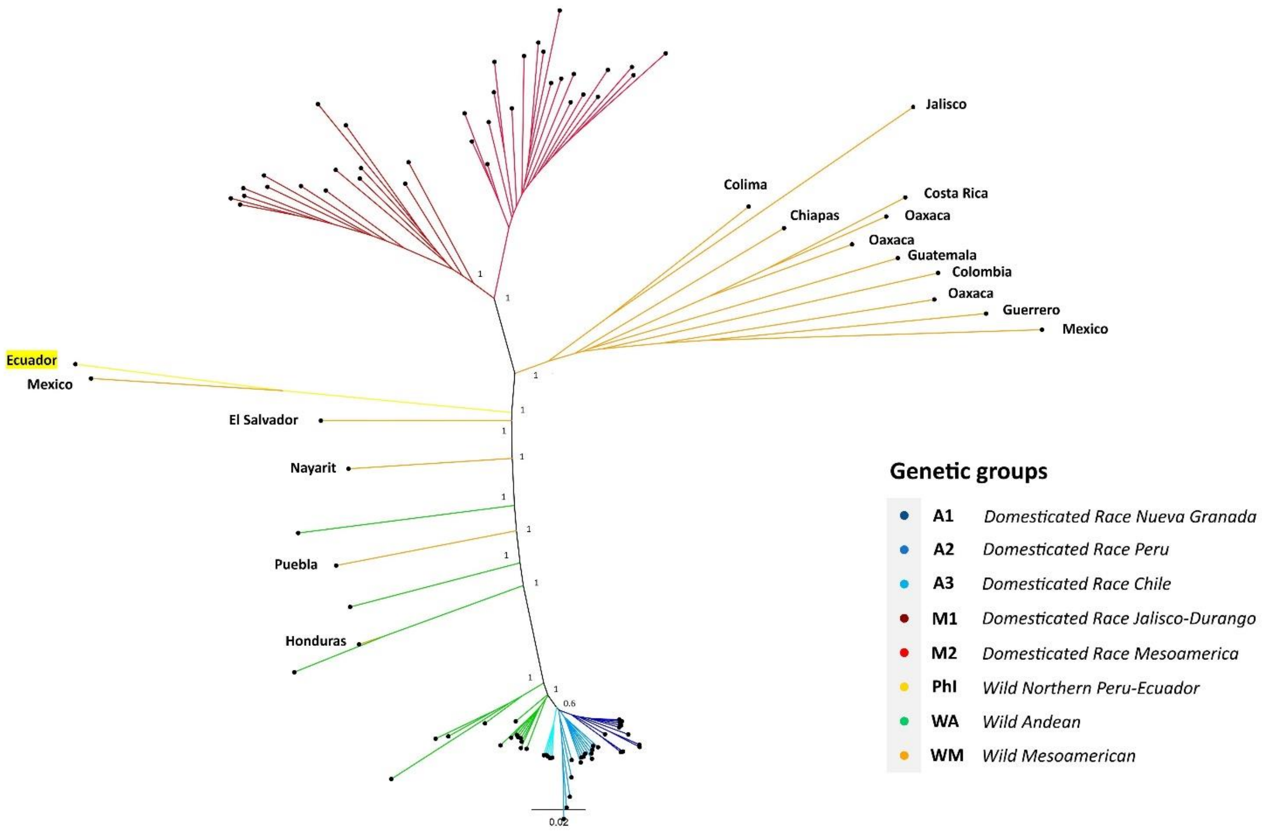

Supplementary Fig. 4. Population structure of *P. vulgaris*. Neighbor-joining (NJ) phylogenetic tree constructed using only SNPs located in PAVs (bootstrap = 1,000).

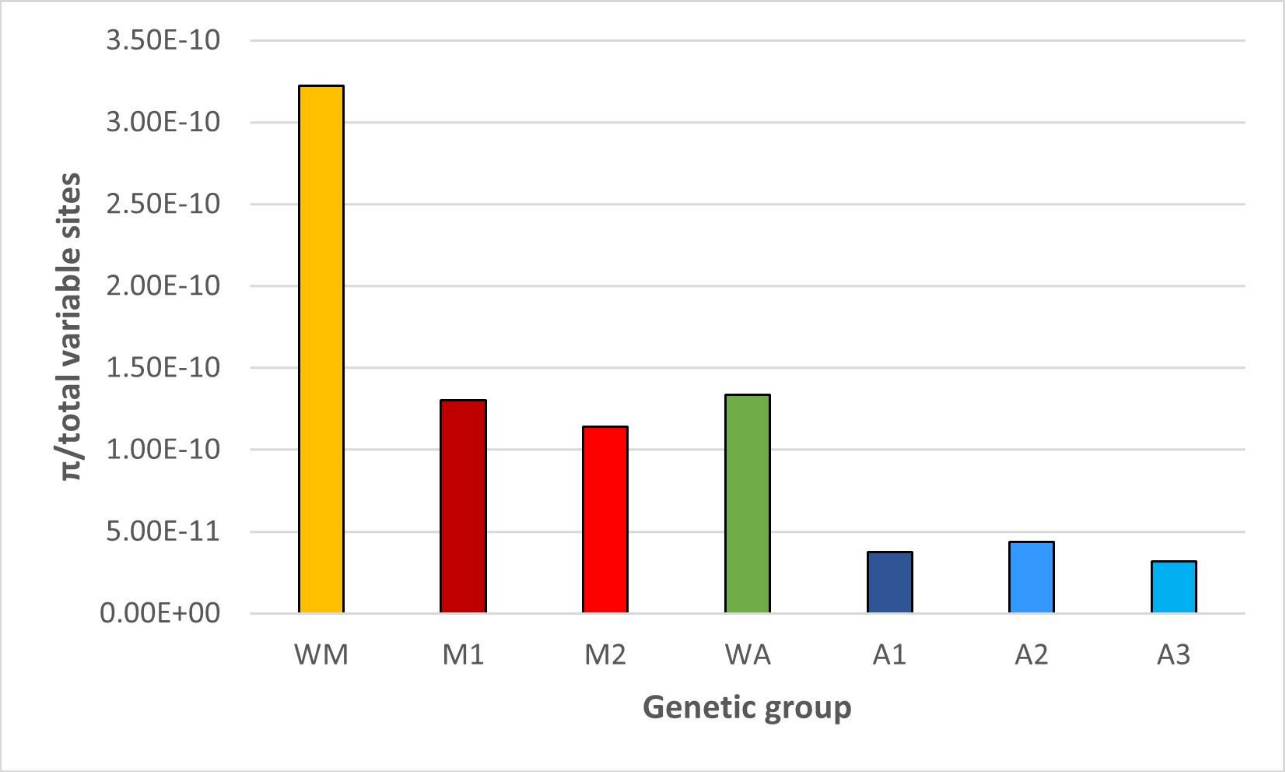

Supplementary Fig. 5. Population structure of *P. vulgaris*. Bar chart showing nucleotide diversity calculated by estimating π in 250-kb windows.

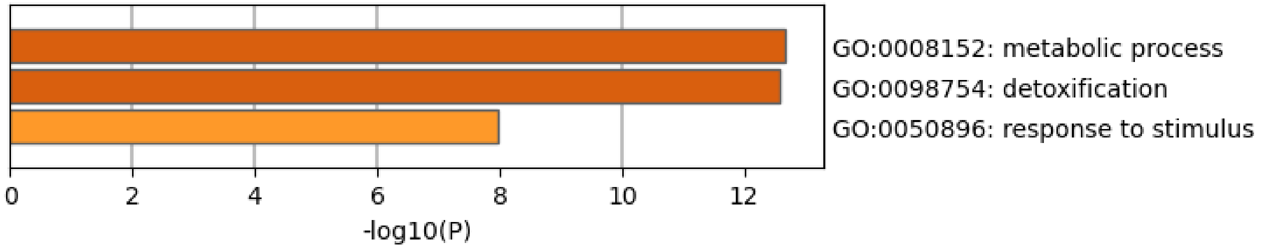

Supplementary Fig. 6. Population structure of *P. vulgaris*. Bar chart showing the GO enrichment analysis of diagnostic genes identified in the American domesticated accessions, specifically comparing the Mesoamerican and Andean gene pools. Source data are provided as a Source Data file.

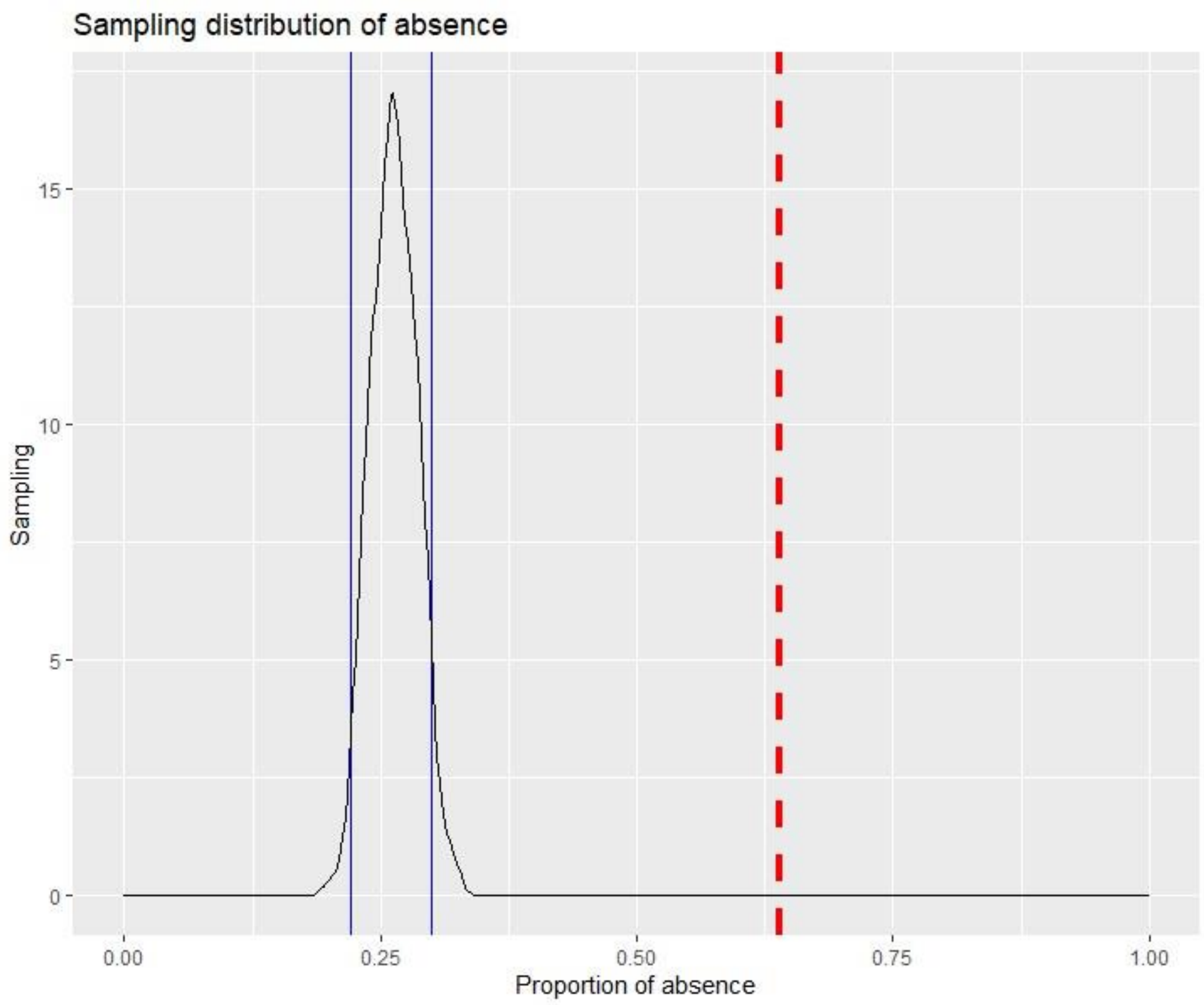

Supplementary Fig. 7. Evolution of the common bean pan-genome. Bootstrap with resampling analysis related to the absence distribution considering the total PAVs in the wild Andean population. The dashed red line corresponds to the ratio of absences putatively under selection to the total PAVs under selection. Source data are provided as a Source Data file.

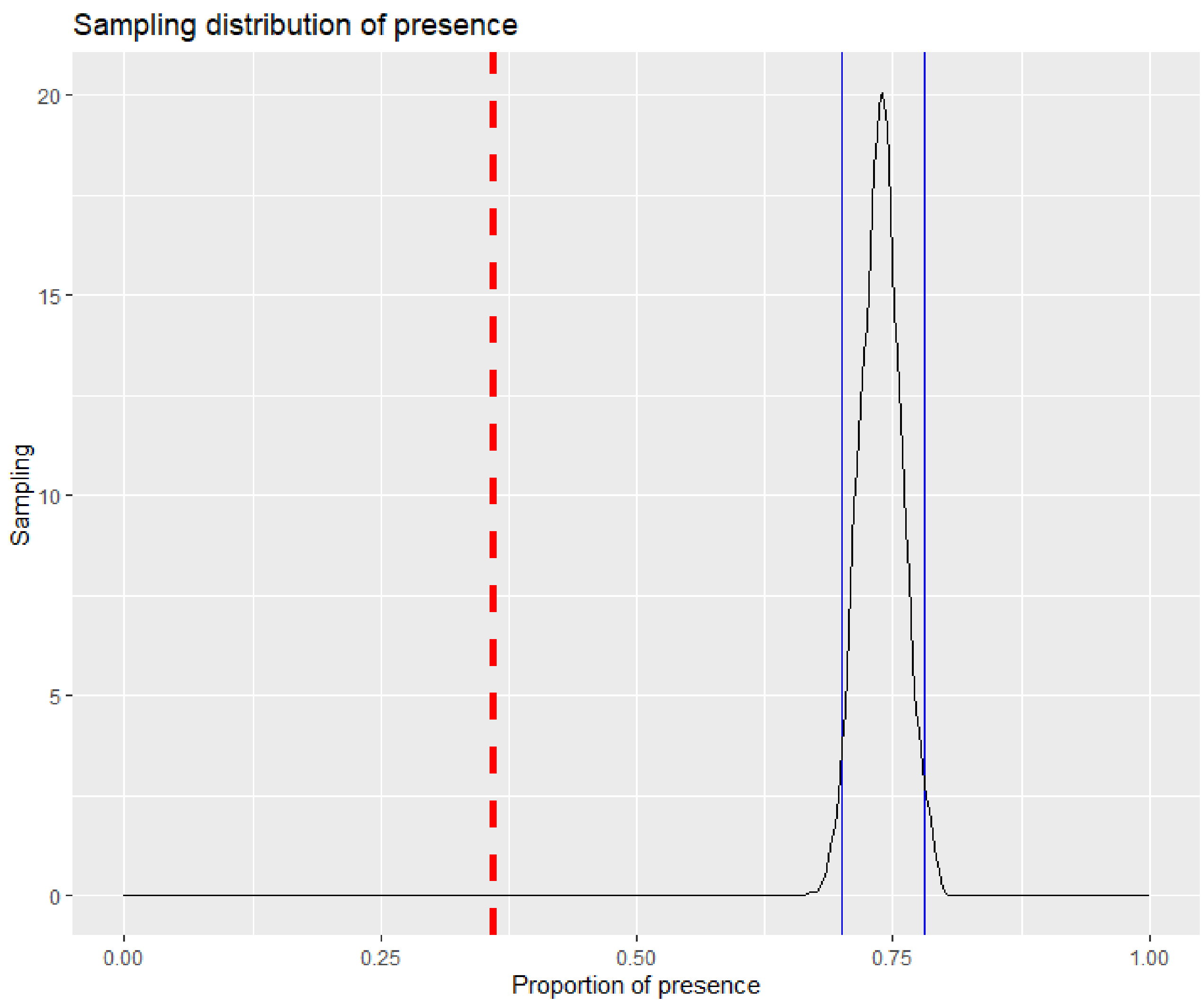

Supplementary Fig. 8. Evolution of the common bean pan-genome. Bootstrap with resampling analysis related to the presence distribution considering the total PAVs in the wild Andean population. The dashed red line corresponds to the ratio of presences putatively under selection to the total PAVs under selection.

Supplementary Table 1. Total length, number of sequences, and number of coding genes annotated at each step during the pan-genome construction, from the initial reference genome to the final pan-genome (G19833 v2.1 + non-reference regions (NRRs) extracted from the four high-quality genomes and from the 339 accessions).

	**G19833 v2.1 reference genome**	**NRRs from MIDAS, G12873, BAT93, and JaloEPP558**	**NRRs from the 339 low coverage accessions**	**Total pangenome**
**Total length (bp)**	537,218,636	97,002,381	136,497,716	770,718,733
**Number of sequences**	478	7,211	54,469	62,158
**Number of protein coding genes**	27,433	2,579	4,326	34,338

Supplementary Table 2. Classification of the pan-genome genes as core or PAV (presence/absence variation).

	**Number of annotated genes**	**CORE genes (%)**	**PAVs (%)**
**Reference G19833 v2.1**	27,433	20,369	7,064
**Non-reference regions - NRRs**	6,905	/	6,905
**Total**	34,338	20,369<br>**59.32%**	13,969<br>**40.68%**

Supplementary Table 3. Two-sided statistic related to the average Ka/Ks value by comparing core vs soft-core vs accessory vs rare.

**Pairwise Wilcoxon test + *p*-value adjustment Benjamini-Hochberg**			
***p*-value**	CORE	SOFTCORE	ACCESSORY
SOFTCORE	< 2.00E-16	-	-
ACCESSORY	< 2.00E-16	0.11471	-
RARE	0.00026	0.0299	0.05492

Supplementary Table 4. Analysis of variance for cluster A and cluster B belonging to the M1/Jalisco-Durango races based on the first principal component of the days to flowering and photoperiod sensitivity as phenotypic trait. Statistical significance was determined by applying a two-sided Student's *t* test.

**Summary of fit**					
Resquare	Adj resquare	Root mean square error	Mean of response	Observations	
0.757248	0.738575	1.580963	6.065333	15	
**Analysis of variance**					
**Source**	**DF**	**Sum of squares**	**Mean square**	**F ratio**	**Prob > F**
Cluster	1	101.35881	101.359	40.5526	<0.0001*
Error	13	32.49276	2.499	-	-
Total	14	133.85157	-	-	-
**Means for One way ANOVA**					
**Level**	**Number**	**Mean**	**Std error**	**Lower 95%**	**Upper 95%**
**cluster A**	7	8.84429	0.59755	7.5534	10.135
**cluster B**	8	3.63375	0.55895	2.4262	4.841
**Means comparison using Student's t**					
t	***Alpha***				
2.16037	0.5				
**Positive values show pairs of means that are significantly different**					
	**cluster A**	**cluster B**			
**cluster A**	-1.82564	3.442866			
**cluster B**	3.442866	-1.70773			

Supplementary Table 5. Two-sided statistic related to the total number of PAVs per genetic group.

**Chi-square test + *p*-value adjustment using false discovery rate (FDR)**							
	**WM**	**M1**	**M2**	**WA**	**A1**	**A2**	**A3**
**WM**	1.00E+00	1.66E-04	2.61E-05	1.60E-11	7.62E-231	6.21E-73	1.54E-161
**M1**	1.66E-04	1.00E+00	6.54E-01	3.15E-03	2.14E-181	2.94E-46	1.96E-120
**M2**	2.61E-05	6.54E-01	1.00E+00	1.18E-02	4.31E-176	1.52E-43	4.98E-116
**WA**	1.60E-11	3.15E-03	1.18E-02	1.00E+00	6.93E-147	1.20E-29	2.19E-92
**A1**	7.62E-231	2.14E-181	4.31E-176	6.93E-147	1.00E+00	4.83E-48	4.96E-08
**A2**	6.21E-73	2.94E-46	1.52E-43	1.20E-29	4.83E-48	1.00E+00	1.01E-19
**A3**	1.54E-161	1.96E-120	4.98E-116	2.19E-92	4.96E-08	1.01E-19	1.00E+00

Supplementary Table 6. Two-sided statistics related to the total number of PAVs per individual.

**Group**	**Number of accessions**	**Mean**	**sd**	**Median**	**IQR**	
<chr>	<int>	<dbl>	<dbl>	<dbl>	<dbl>	
**A1**	11	6432	223	6413	332	
**A2**	14	6624	152	6638	162	
**A3**	5	6499	160	6554	160	
**M1**	15	8818	148	8830	196	
**M2**	21	8799	211	8813	274	
**WA**	16	6792	531	6605	382	
**WM**	16	8306	525	8430	476	
**Pairwise Wilcoxon test + *p*-value adjustment Benjamini-Hochberg**						
	**WM**	**M1**	**M2**	**WA**	**A1**	**A2**
**M1**	7.80E-05	-	-	-	-	-
**M2**	0.00045	0.88969	-	-	-	-
**WA**	1.90E-06	1.40E-07	9.90E-07	-	-	-
**A1**	8.10E-07	9.90E-07	1.20E-05	0.04439	-	-
**A2**	1.40E-07	1.80E-07	2.10E-06	0.95104	0.04014	-
**A3**	0.00019	0.00023	0.00107	0.36487	0.48735	0.2406
**Tukey-Kramer HSD**						
**Genetic group**	**Level**	**Mean**				
**M1**	A	8818.13				
**M2**	A	8799.24				
**WM**	B	8305.81				
**WA**	C	6792.25				
**A2**	C	6624.07				
**A3**	C	6499.2				
**A1**	C	6432				

Supplementary Table 7. Analysis of variance related to the total number of PAVs per individual by geographical groups related to the wild accessions. Statistical significance was determined by applying a two-sided Tukey-Kramer HSD test.

**Summary of fit**					
Resquare	Adj resquare	Root mean square error	Mean of response	Observations	
0.69	0.66	536.86	6365.61	31	
**Analysis of variance**					
**Source**	**DF**	**Sum of squares**	**Mean square**	**F ratio**	**Prob > F**
Group	3	17578010	5859337	20.3297	<.0001*
Error	27	7781833	288216	-	-
Total	30	25359843	-	-	-
**Tukey-Kramer HSD**					
**Level**	**Letter**	**Mean**			
Group I	A	7.13E+03			
Group II	A	6.99E+03			
Group III	B	5.79E+03			
Group IV	B	5.47E+03			

Supplementary Table 8. Gene frequency of the 29 PAVs with high FST values between wild and domesticated forms common between the two gene pools (Mesoamerican and Andean).

PAVs	Freq. wild Mesoamerican	Freq. domesticated Mesoamerican	Freq. wild Andean	Freq. domesticated Andean
g19607	1.00	0.81	1.00	0.60
g22208	1.00	0.83	1.00	0.63
g22667	1.00	0.83	1.00	0.63
g19935	1.00	0.86	1.00	0.67
g20101	1.00	0.86	1.00	0.70
g20268	1.00	0.69	1.00	0.70
g19351	1.00	0.83	1.00	0.73
g21500	1.00	0.86	1.00	0.73
g22820	1.00	0.86	1.00	0.77
g20407	1.00	0.83	1.00	0.77
g19778	1.00	0.83	1.00	0.80
g19917	1.00	0.81	1.00	0.80
g20866	1.00	0.86	1.00	0.80
g25047	1.00	0.86	1.00	0.80
Phvul.L001890	1.00	0.78	1.00	0.83
g19697	1.00	0.86	1.00	0.83
g20642	1.00	0.81	1.00	0.83
g20836	1.00	0.86	1.00	0.83
g19911	1.00	0.86	1.00	0.87
g20485	1.00	0.83	1.00	0.87
g20849	1.00	0.86	1.00	0.87
Phvul.005G018900	0.88	0.36	1.00	0.77
a6801	0.81	0.19	1.00	0.63
Phvul.006G092700	0.75	0.06	1.00	0.60
Phvul.005G066300	0.75	0.11	0.81	0.07
Phvul.008G179700	0.25	0.89	0.13	0.90
g16398	0.25	0.92	0.00	0.37
g39977	0.13	0.67	0.00	0.20
g21440	0.06	0.94	0.13	0.83

Supplementary Table 9. Assembly statistics related to the five *P. vulgaris* genomes used to construct the pan-genome.

	G19833 v2.1<br>Reference	MIDAS	G12873	BAT93	JALOEPP558
	**Domesticated Andean**	**Domesticated Andean**	**Wild Mesoamerican**	**Domesticated Mesoamerican**	**Domesticated Andean**
**Total assembly size (bp)**	537,218,636	508,765,391	584,634,161	627,259,432	576,418,805
**Number of scaffolds**	478	-	-	-	-
**Number of contigs**	1,044	1,878	6,173	1,153	699
**Contigs average length (bp)**	509,156	270,908	94,708	544,024	824,633
**Contigs N50 (bp)**	1,885,876	3,412,857	2,176,347	11,017,447	14,208,857
**Contigs N90 (bp)**	377,857	212,756	57,629	1,559,241	3,218,403
**Longest contigs (bp)**	12,554,793	24,636,533	20,321,960	36,599,242	45,469,680

Supplementary Table 10. Assembly statistics related to the MIDAS and G12873 genomes.

	MIDAS - domesticated Andean	G12873 – wild Mesoamerican
**Total assembly size (bp)**	508,765,391	584,634,161
**Number of contigs**	1,878	6,173
**Contigs average length (bp)**	270,908	94,708
**Contigs N50 (bp)**	3,412,857	2,176,347
**Contigs N90 (bp)**	212,756	57,629
**Longest contigs (bp)**	24,636,533	20,321,960
**BUSCO completeness**	C:95.8%[S:94.4%,D:1.4%],F:0.8%,M:3.8%	C:94.6%[S:92.7%,D:1.9%],F:0.4%,M:5.0%
