# Supplementary material for: Adaptive gene loss in the common bean pan-genome during range expansion and domestication
Source: Nat Commun. 2024 Aug 7;15:6698. doi: 10.1038/s41467-024-51032-2 (PMC11303546; doi:10.1038/s41467-024-51032-2)
Supplement: Supplementary file 3 — Description of Additional Supplementary Files [file 41467_2024_51032_MOESM3_ESM.pdf]

File Name: Supplementary Data 1

Description: Presence/absence scores of each predicted pan-genome gene for each accession (1 = present, 0 = absent).

File Name: Supplementary Data 2

Description: GO enrichment analysis performed using the enricher function from the clusterProfiler R package, which employs a hypergeometric test to identify over-represented GO terms in the core genes. To control for multiple comparisons, the p-values were adjusted using the Benjamini-Hochberg (BH) method.

File Name: Supplementary Data 3

Description: GO enrichment analysis performed using the enricher function from the clusterProfiler R package, which employs a hypergeometric test to identify over-represented GO terms in the PAV genes. To control for multiple comparisons, the p-values were adjusted using the Benjamini-Hochberg (BH) method.

File Name: Supplementary Data 4

Description: Calculation of the non-synonymous/synonymous ratio (Ka/Ks) for each gene in each accession and the corresponding average value (GP column) used for the subsequent statistical analysis. The Ka/Ks analysis was applied only to the representative panel of 99 accessions.

File Name: Supplementary Data 5

Description: Estimation of the nucleotide diversity (π) per genetic group in genome-wide 250-kb windows.

File Name: Supplementary Data 6

Description: Diagnostic American PAVs identified by applying Fisher's exact test (two-sided) between the Mesoamerican and Andean accessions. The frequencies of diagnostic American PAVs in the European accessions are shown in red columns.

File Name: Supplementary Data 7

Description: Fisher's exact test (two-sided) between the Mesoamerican and Andean accessions based on the European panel.

File Name: Supplementary Data 8

Description: List of presence-absence variations (PAVs) identified with PAV-based GWAS demonstrating significant associations with flowering time and photoperiod sensitivity (DTF; See Bellucci et al., 2023 for phenotypic data information). The GWAS analysis was conducted using two statistical models: the Mixed Linear Model (MLM) and the Fixed and Random Model Circulating Probability Unification (FarmCPU) model. The threshold for each scan was determined by the Bonferroni corrected p value at $\alpha = 0.05$ ($p \leq 7.07E-06$).

File Name: Supplementary Data 9

Description: Calculation of FST values (column AT) for each PAV between wild Mesoamerican and wild Andean gene pools. To optimize the analysis, 7 wild Mesoamerican and Andean genotypes that clustered togheter in the NJ-tree (503MW_A, 510MW_B, 65, 790, 38, 67_AW_A, 716) were excluded.

File Name: Supplementary Data 10

Description: Manual gene function investigation for the 402 PAVs with high FST values (FST ≥ 0.85) between wild Mesoamerican and wild Andean populations.

File Name: Supplementary Data 11

Description: Calculation of FST values (column BU) for each PAV between wild and domesticated forms belonging to the Mesoamerican gene pool.

File Name: Supplementary Data 12

Description: Calculation of FST values (column BO) for each PAV between wild and domesticated forms belonging to the Andean gene pool.

File Name: Supplementary Data 13

Description: Manual gene function investigation for the 610 PAVs with high FST values (FST ≥ 0.30) between wild and domesticated forms belonging to the Mesoamerican gene pool.

File Name: Supplementary Data 14

Description: Manual gene function investigation for the 497 PAVs with high FST values (FST ≥ 0.27) between wild and domesticated forms belonging to the Andean gene pool.

File Name: Supplementary Data 15

Description: Information related to the 339 accessions used to construct the common bean pan-genome. Yellow highlights indicate a subset of 99 accessions comprising American wild and domesticated forms representing the main subset for data analysis. Green highlights indicate a subset of 114 European domesticated accessions used for part of the data analysis.

File Name: Supplementary Data 16

Description: Identity percentages between the open reading frames of 2,330 complete single-copy BUSCO genes and MIDAS and G12873 to determine orthologous sequence similarities.

File Name: Supplementary Data 17

Description: Identity percentages in three gene families based on all-versus-all comparison between the members of the same family to observe paralogous sequences similarities.

File Name: Supplementary Data 18

Description: Table of the sequencing coverages for each gene in each accession.

File Name: Supplementary Data 19

Description: Calculation of the pan-genes growth.

File Name: Supplementary Data 20

Description: Calculation of the core-genes growth.
