# Supplementary material for: Adaptive gene loss in the common bean pan-genome during range expansion and domestication
Source: Nat Commun. 2024 Aug 7;15:6698. doi: 10.1038/s41467-024-51032-2 (PMC11303546; doi:10.1038/s41467-024-51032-2)
Supplement: Supplementary file 24 — Reporting Summary [file 41467_2024_51032_MOESM24_ESM.pdf]

nature portfolio

Corresponding author(s): Prof. Roberto Papa

Last updated by author(s): 9 July, 2024

Reporting Summary

Nature Portfolio wishes to improve the reproducibility of the work that we publish. This form provides structure for consistency and transparency in reporting. For further information on Nature Portfolio policies, see our Editorial Policies and the Editorial Policy Checklist.

Statistics

For all statistical analyses, confirm that the following items are present in the figure legend, table legend, main text, or Methods section.

n/a	Confirmed	
☐	☒	The exact sample size (*n*) for each experimental group/condition, given as a discrete number and unit of measurement
☒	☐	A statement on whether measurements were taken from distinct samples or whether the same sample was measured repeatedly
☐	☒	The statistical test(s) used AND whether they are one- or two-sided<br>*Only common tests should be described solely by name; describe more complex techniques in the Methods section.*
☒	☐	A description of all covariates tested
☐	☒	A description of any assumptions or corrections, such as tests of normality and adjustment for multiple comparisons
☐	☒	A full description of the statistical parameters including central tendency (e.g. means) or other basic estimates (e.g. regression coefficient) AND variation (e.g. standard deviation) or associated estimates of uncertainty (e.g. confidence intervals)
☐	☒	For null hypothesis testing, the test statistic (e.g. *F*, *t*, *r*) with confidence intervals, effect sizes, degrees of freedom and *P* value noted<br>*Give P values as exact values whenever suitable.*
☒	☐	For Bayesian analysis, information on the choice of priors and Markov chain Monte Carlo settings
☒	☐	For hierarchical and complex designs, identification of the appropriate level for tests and full reporting of outcomes
☒	☐	Estimates of effect sizes (e.g. Cohen's *d*, Pearson's *r*), indicating how they were calculated

Our web collection on statistics for biologists contains articles on many of the points above.

Software and code

Policy information about availability of computer code

Data collection	A detailed description of the sofwares/packages/tools used for data collection are described in the Methods section. Here is a summary: Phytozome, NanoDrop 1000 spectrophotometer (Thermo Fisher Scientific), Qubit 4.0 (Thermo Fisher Scientific), CHEF Mapper electrophoresis system (Bio-Rad Laboratories), Short Reads Eliminator kit (Circulomics), DNeasy 96 Plant kit (Qiagen), Tissue Lyser II (Qiagen), NanoPhotometer NP80 (Implen), KAPA Hyper Prep kit and PCR-free protocol (Roche), Covaris S220 device (Covaris), AMPure XP beads, KAPA Library Quantification Kit (Kapa Biosystems), NovaSeq 6000 (Illumina), MinION device with a SpotON flow cell (FLO-MIN106 R9.4.1 Rev D) (ONT), SQK-LSK109 ligation sequencing protocol (ONT), Flow Cell Wash Kit (ONT), Bioanalyzer 2100, Canu v2.1, wtdbg2 v2.5, Racon v1.4.3, Medaka v1.0.3, Pilon v1.23, BUSCO v4.1.2, HiFiasm v0.9.0, minimap2 v2.17, samtools depth v1.1, Assemblytics v1.2.1, CD-HIT-EST v4.8.1, BLASTp, fastp v0.21.0, bowtie2 v2.3.5.1, MaSuRCA v3.4.2, BLASTn, RepeatModeler v2.0.2, RepeatMasker v4.1.2, Augustus v3.3.3, Hisat2 v2.2.1, Genome Threader v1.7.1, InterProScan v5.46, OrthoFinder v2.5.4, custom script, bcftools v1.10.2.
Data analysis	A detailed description of the software/packages/tools used for data analysis is provided in the Methods section under 'Data Analysis'. Here is a summary: buildGOmap R function, clusterProfiler, Metascape, OrthoFinder, logisticPCA package in R, bcftools consensus v1.10.2, KaKs calculator v2.0, R, vcftools, MEGA11, FigTree, Tassel 5, gstat package in R, QGIS, GAPIT v3.

For manuscripts utilizing custom algorithms or software that are central to the research but not yet described in published literature, software must be made available to editors and reviewers. We strongly encourage code deposition in a community repository (e.g. GitHub). See the Nature Portfolio guidelines for submitting code & software for further information.

Data

Policy information about availability of data

All manuscripts must include a data availability statement. This statement should provide the following information, where applicable:

- Accession codes, unique identifiers, or web links for publicly available datasets
- A description of any restrictions on data availability
- For clinical datasets or third party data, please ensure that the statement adheres to our policy

The 109 raw WGS reads generated in this study have been deposited in the National Center of Biotechnology Information (NCBI) Sequence Read Archive (SRA) under BioProject number PRJNA1042929 [https://www.ncbi.nlm.nih.gov/bioproject/PRJNA1042929]. Additional WGS data comprising 10 and 220 raw WGS reads were sourced from BioProject numbers PRJNA910538 [https://www.ncbi.nlm.nih.gov/bioproject/PRJNA910538/] and PRJNA573595 [https://www.ncbi.nlm.nih.gov/bioproject/PRJNA573595], respectively. The RNA-Seq data from this study have been deposited in the SRA under BioProject number PRJNA1042929 [https://www.ncbi.nlm.nih.gov/bioproject/PRJNA1042929]. Additionally, 21 RNA samples were sourced from BioProject number PRJNA212729 [https://www.ncbi.nlm.nih.gov/bioproject/PRJNA212729]. The reference genome G19833 v2.1 is available in Phytozome at: [https://phytozome-next.jgi.doe.gov/info/Pvulgaris_v2_1]. The other four high-quality genomes have been deposited in the National Center of Biotechnology Information (NCBI) Sequence Read Archive (SRA) under BioProject number PRJNA1042929 [https://www.ncbi.nlm.nih.gov/bioproject/PRJNA1042929]. The pan-genome assembly and its annotation have been deposited in Figshare and are available at: https://doi.org/10.6084/m9.figshare.24573874. Source data are provided with this paper. Moreover, the custom codes used in this study have been deposited on GitHub and are available at the following link: https://doi.org/10.5281/zenodo.12191159.

Research involving human participants, their data, or biological material

Policy information about studies with human participants or human data. See also policy information about sex, gender (identity/presentation), and sexual orientation and race, ethnicity and racism.

Reporting on sex and gender	N/A
Reporting on race, ethnicity, or other socially relevant groupings	N/A
Population characteristics	N/A
Recruitment	N/A
Ethics oversight	N/A

Note that full information on the approval of the study protocol must also be provided in the manuscript.

Field-specific reporting

Please select the one below that is the best fit for your research. If you are not sure, read the appropriate sections before making your selection.

[x] Life sciences [ ] Behavioural & social sciences [ ] Ecological, evolutionary & environmental sciences

For a reference copy of the document with all sections, see nature.com/documents/nr-reporting-summary-flat.pdf

Life sciences study design

All studies must disclose on these points even when the disclosure is negative.

Sample size	The sample size for this study was chosen to comprehensively capture the extensive genetic variation within Phaseolus vulgaris. Our pan-genome construction included both wild and domesticated accessions, totaling 339 genotypes (domesticated n=306, wild n=33). The selection process considered various factors including gene pool, representation of genetic subgroups, geographic origin, chloroplast inversion patterns, response to flowering time, and biological status. These factors were critical to achieving a comprehensive and representative sampling of the genetic diversity within the species.
Data exclusions	Out of the 339 genotypes used for pan-genome construction, our data analysis focused on a representative panel of 99 genotypes that have been well characterized phenotypically and genetically in previous studies. We excluded common bean accessions with limited information to ensure robust insights into genetic variation in terms of PAVs and evolutionary patterns.
Replication	We did not conduct phenotypic trials with replicates. The experimental design focused on pan-genome construction and genomic analyses, where reproducibility was ensured through rigorous bioinformatic and statistical methods applied to the genomic data.
Randomization	Randomization was not relevant to our study because we did not collect phenotypic data.
Blinding	Blinded data were not relevant to our study because we aimed to analyze all presence/absence variations.

Reporting for specific materials, systems and methods

We require information from authors about some types of materials, experimental systems and methods used in many studies. Here, indicate whether each material, system or method listed is relevant to your study. If you are not sure if a list item applies to your research, read the appropriate section before selecting a response.

Materials & experimental systems

n/a	Involved in the study
☒	☐ Antibodies
☒	☐ Eukaryotic cell lines
☒	☐ Palaeontology and archaeology
☒	☐ Animals and other organisms
☒	☐ Clinical data
☒	☐ Dual use research of concern
☐	☒ Plants

Methods

n/a	Involved in the study
☒	☐ ChIP-seq
☒	☐ Flow cytometry
☒	☐ MRI-based neuroimaging

Dual use research of concern

Policy information about dual use research of concern

Hazards

Could the accidental, deliberate or reckless misuse of agents or technologies generated in the work, or the application of information presented in the manuscript, pose a threat to:

No	Yes
☒	☐ Public health
☒	☐ National security
☒	☐ Crops and/or livestock
☒	☐ Ecosystems
☒	☐ Any other significant area

Experiments of concern

Does the work involve any of these experiments of concern:

No	Yes
☒	☐ Demonstrate how to render a vaccine ineffective
☒	☐ Confer resistance to therapeutically useful antibiotics or antiviral agents
☒	☐ Enhance the virulence of a pathogen or render a nonpathogen virulent
☒	☐ Increase transmissibility of a pathogen
☒	☐ Alter the host range of a pathogen
☒	☐ Enable evasion of diagnostic/detection modalities
☒	☐ Enable the weaponization of a biological agent or toxin
☒	☐ Any other potentially harmful combination of experiments and agents

Plants

Seed stocks	Information regarding the seed stocks related to the common bean genotypes used in this study is reported in Supplementary Data 14.
Novel plant genotypes	No novel plant genotypes have been developed in this study.
Authentication	N/A
